# Niosomes: A Strategy toward Prevention of Clinically Significant Drug Incompatibilities

**DOI:** 10.1038/s41598-017-06955-w

**Published:** 2017-07-24

**Authors:** Hebatallah B. Mohamed, Sohair M. El-Shanawany, Mostafa A. Hamad, Mahmoud Elsabahy

**Affiliations:** 1 Department of Pharmaceutics, Faculty of Pharmacy, Assiut University, Assiut, 71515 Egypt; 2 Department of Surgery, Faculty of Medicine, Assiut University, Assiut, Egypt; 3 Assiut International Center of Nanomedicine, Al-Rajhy Liver Hospital, Assiut University, Assiut, Egypt; 4 Laboratory for Synthetic-Biologic Interactions, Department of Chemistry, Texas A&M University, College Station, Texas, USA; 5 Misr University for Science and Technology, 6th of October City, Giza, Egypt

## Abstract

Drug incompatibilities are considered as one of the most critical problems in intensive care units. In the current study, the ability of nanomaterials to prevent drug incompatibilities in clinical settings has been investigated. As a proof-of-concept, the ability of niosomes to prevent physical and chemical incompatibilities that occur upon mixing acyclovir and vancomycin during management of acute meningitis has been explored. Nanosized spherical particles loaded separately with either vancomycin or acyclovir, with high entrapment efficiency (*ca*. 46–56%), could be prepared, and sustained release of their entrapped cargoes have been demonstrated over time. We have shown that precipitation, degradation and loss of biological activity of drugs occurred upon mixing solutions of the free drugs. On the contrary, drugs loaded separately inside niosomal structures exhibited high stability, exceptional physical and chemical compatibilities for up to 48 h with complete preservation of the antimicrobial activity of vancomycin. This study opens a venue for a new spectrum of applications of nanomaterials in preventing clinically significant drug incompatibilities, aiming at the reduction of adverse reactions, cost and hospitalization period, and improvement of patient compliance and therapeutic outcomes.

## Introduction

Drug incompatibilities are considered as one of the most critical problems in intensive care units^1–4^. Patients in intensive care units receive several intravenous solutions sequentially in the same vein through the same cannula or *via* mixing two or more parenteral solutions into the same infusion bag, which increase the risk of physicochemical incompatibilities. Physical incompatibility in the form of drug precipitation is one of the common incompatibilities that result in formation of particles in the infusion lines, usually due to the change in pH or buffering capacity of the solution. Chemical degradation and decomposition can also be accelerated after precipitation, where degradation of drugs reduces the amount of active drug and also may lead to the formation of toxic by-products. Physical and chemical incompatibilities may cause thrombophlebitis, pulmonary embolism and tissue irritation, and result in therapeutic failure. These adverse effects are usually manifested in pediatrics and neonates. In addition, these incompatibilities reduce patient compliance and increase the total cost of medication and extend hospitalization time.

For instance, physicochemical incompatibilities occur upon mixing intravenous solutions of the antibacterial drug, vancomycin, and the antiviral drug, acyclovir. Combinational therapy between vancomycin and acyclovir is beneficial in the management of meningitis^5^. because both bacterial and viral infections have the same symptoms and can be differentiated only *via* withdrawal of cerebrospinal fluid (a lumber puncture), which is usually performed with high caution and under aseptic conditions, and contraindicated in some cases (*e*.*g*. bleeding disorders and high intracranial pressure)^6^. Acyclovir is available as acyclovir sodium salt for intravenous infusion with reconstituted solution pH of 11. Vancomycin has broad spectrum bactericidal activity on Gram positive bacteria that may cause meningitis (*e*.*g*. *Staphylococcus aureus* and other methicillin-resistant *Staphylococcus* species)^5^, and it is available as vancomycin hydrochloride for intravenous infusion with reconstituted solution pH in the range of 2.8–4.5. Mixing solutions that contain high concentrations of both drugs is important to lower the volume of the administered solution, and, thus lowering the susceptibility to brain edema^7^. However, each drug is usually given *via* separate infusion because mixing of the two parenteral solutions that have different pHs in the same infusion bag results in precipitation due to changes in the ionization degrees of the drugs. In addition, at high concentrations and at neutral pH, both drugs (either separate or mixed) can precipitate and/or degrade over time^8, 9^. Furthermore, repeated administration of the drugs is usually necessary due to their short elimination half-lives *in vivo*.

Nanocarriers play an important role in protecting drugs from degradation and in delivering them to their target sites, and have been extensively utilized for various biomedical applications^10–13^. However, nanomaterials have not been tested previously for their ability to prevent drug incompatibilities. As a proof-of-concept, the ability of nanomaterials (*i*.*e*. niosomes) to prevent incompatibilities between two drugs (*i*.*e*. acyclovir and vancomycin) that are commonly utilized *via* intravenous infusion for treatment of acute meningitis has been explored. In the current study, we took the advantages of nanomaterials in isolating drugs from the surrounding medium to prevent the physicochemical incompatibilities between vancomycin and acyclovir by loading the drugs separately into niosomes. Hence, both drugs can be given at high concentrations in small volumes of parenteral solutions that are prepared at pH mimics the physiological pH (*i*.*e*. pH 7.4). In addition, sustained release of the entrapped drugs from nanocarriers over time can protect drugs from degradation and reduce the frequency of administration.

## Results

Parenteral administration is a potential source of incompatibilities between drugs in intensive care units. When drugs are mixed together in the same infusion bag, physical and chemical incompatibilities can become a serious problem that lead to severe adverse reactions and complications. Physical incompatibilities are easily identifiable in the form of precipitation, turbidity, color change, *etc*. On the other hand, chemical incompatibilities should be carefully assessed as it may produce toxic or inactive degradation products that may not be detected visually. Most of parenteral drugs are organic weak electrolytes in predominantly ionized or salt forms, and, thus precipitation-based incompatibility frequently occurs due to alteration of pH upon mixing. Introduction of these precipitates to the systemic circulation reduces the amount of active drug available, and can induce phlebitis and pulmonary embolism.

Management of acute meningitis requires a combination between vancomycin and acyclovir. Acyclovir has antiviral activity against several viruses and it has poor oral bioavailability of *ca*. 15–20%. The reconstituted vial (1 g/20 mL or 50 mg/mL) has a pH of 11. Vancomycin is used in the treatment of serious, life-threatening infections caused by Gram-positive bacteria (*e*.*g*. *Staphylococcus aureus*) that do not respond to other antibiotics. For systemic therapy, it must be given intravenously because it is a large hydrophilic molecule that is poorly partitioned through the gastrointestinal mucosa. Vancomycin solution has a low pH (2.5–4.5), where chemical and/or physical instabilities occur upon mixing with solutions of different pHs. Dilution of vancomycin or acyclovir vials results in a slight change of the solution pH. The highest concentrations of vancomycin and acyclovir in the intravenous infusions range from 5–7 mg/mL, where side effects may occur upon administration of higher concentrations of the free drugs^14–16^.

**Figure 1:**
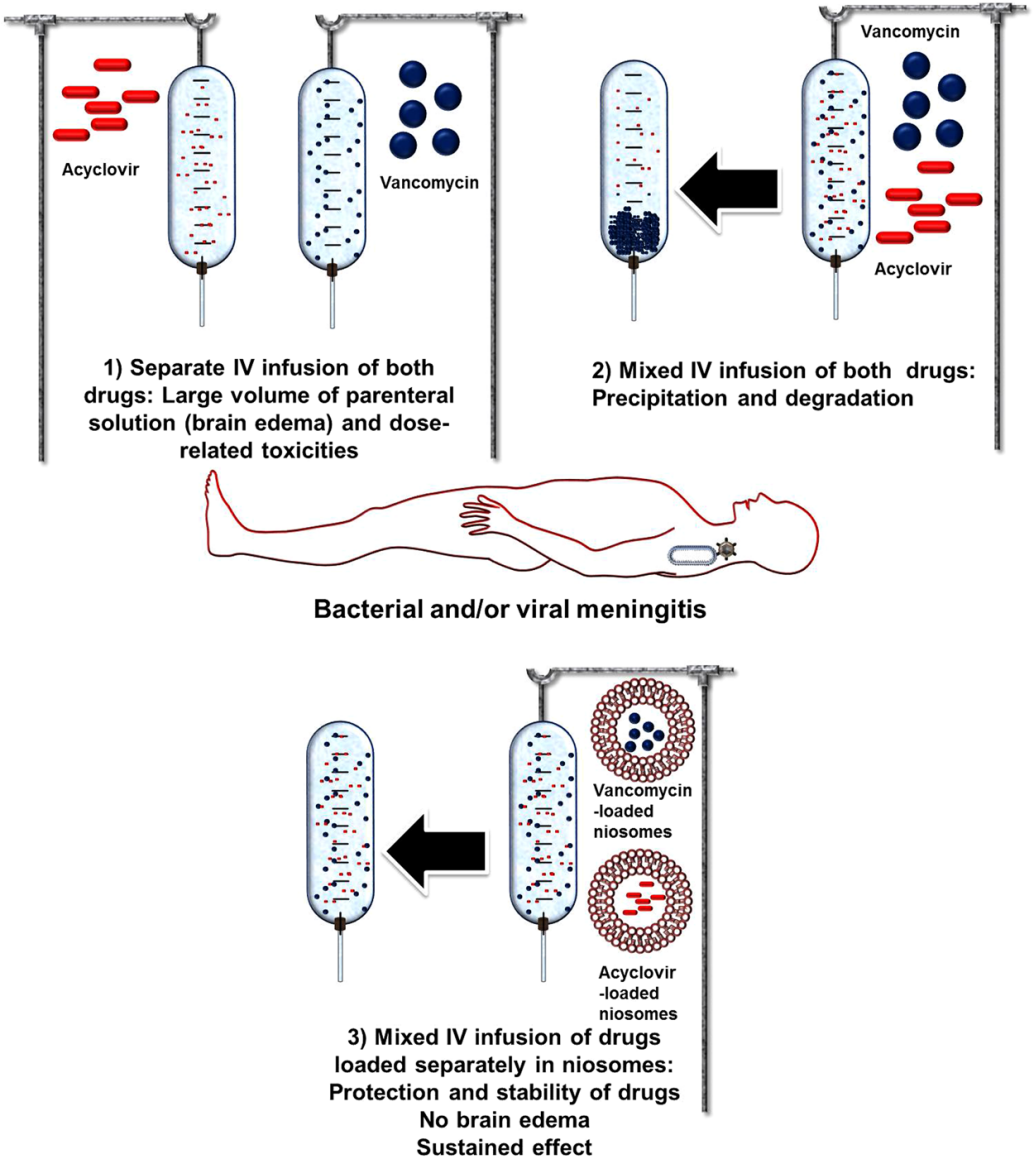
Diagram illustrates the benefits of exploiting niosomes in preventing drug incompatibilities in clinical settings.

Separate infusion of both drugs requires large volume that can lead to brain edema, and also repeated high dosing due to the short circulation time of acyclovir and vancomycin (The elimination half-lives of vancomycin and acyclovir are 4–6 h and 2.5–3 h in subjects with normal renal functions, respectively). Reconstituted solutions (2–7 mg/mL) of vancomycin and acyclovir have pHs of 5.5–6.5 and 9–11.2, respectively (Supplementary Information, Tables S1). Mixing both solutions in the same infusion bag results in precipitation of vancomycin (pH of the mixed solution is 8–10.5), and also degradation of both drugs over time. We proposed that nanoparticulates loaded separately with vancomycin and acyclovir would isolate the drugs within the niosomal nanostructures and allow the preparation of solutions of small infusion volumes of appropriate pH (close to neutrality) in order to prevent precipitation and degradation of drugs and also control the release of the drugs over time, aiming at reducing the frequency of administration and improving patient compliance (Fig. 1). In addition, higher concentrations (*i*.*e*. >5–7 mg/mL) might be given if the drugs exist as reservoirs that are entrapped into the nanocarriers and being released slowly over time.

### Characterizations of the drug-loaded niosomes

Niosomes are formed of bilayer vesicles of nonionic surfactants and cholesterol, and have shown greater physicochemical stability and lower cost of production, as compared to liposomes^17^. Niosomes are considered as effective and stable nanocarriers for encapsulation and delivery of hydrophilic drugs, such as, acyclovir sodium and vancomycin hydrochloride. In the current study, niosomes were prepared from cholesterol, Span^®^ 60 and Tween^®^ 40 nonionic surfactants at equimolar ratio of cholesterol and the nonionic surfactants. Entrapment efficiencies of vancomycin hydrochloride and acyclovir sodium into niosomes were 46.3% and 56.4%, respectively (Table 1). High entrapment efficiency of the water soluble drugs in niosomes is due to the equimolar ratio of cholesterol and the nonionic surfactants and the possible hydrogen bonding between the surfactants and the entrapped drugs^18–22^. The use of high amount of cholesterol results in formation of rigid vesicles with sufficient entrapment of the drugs and sustained release of drugs over time^18, 21^. Span^®^ 60 has a long alkyl chain (C_18_) and high transition temperature (56–58 °C) and strongly interacts with cholesterol molecules, and thus, forming large core space for drug entrapment^18^. It has been also reported that Tween^®^ 40 increases encapsulation of hydrophilic drugs in co-surfactant niosomal formulations^20^. Acyclovir has 2 and 6 hydrogen bond donors and acceptors, respectively, whereas, vancomycin has 20 and 26 hydrogen bond donors and acceptors, respectively^23, 24^. Hence, vancomycin might have greater ability to form hydrogen bonds with the surfactants. However, the higher entrapment efficiency of acyclovir in niosomes might be due to the lower molecular weight of acyclovir as compared to vancomycin (225.2 *vs*. 1449.3 g/mol, respectively).

**Table 1:** Characterization of the niosomal formulations (averaged-number hydrodynamic diameters, polydispersity indices (PDI), Zeta-potential values and encapsulation efficiencies (EE, %)) utilized to prevent incompatibilities between acyclovir sodium and vancomycin hydrochloride (mean ± SD, n = 3).

Sample	Size (nm)	PDI	Zeta-potential (mV)	EE%
Acyclovir-loaded niosomes	89.7 ± 35	0.47 ± 0.12	−46.7 ± 1.5	56.4% ± 1.8
Vancomycin-loaded niosomes	53.7 ± 12	0.45 ± 0.01	−21.6 ± 0.8	46.3% ± 1.1

**Figure 2:**
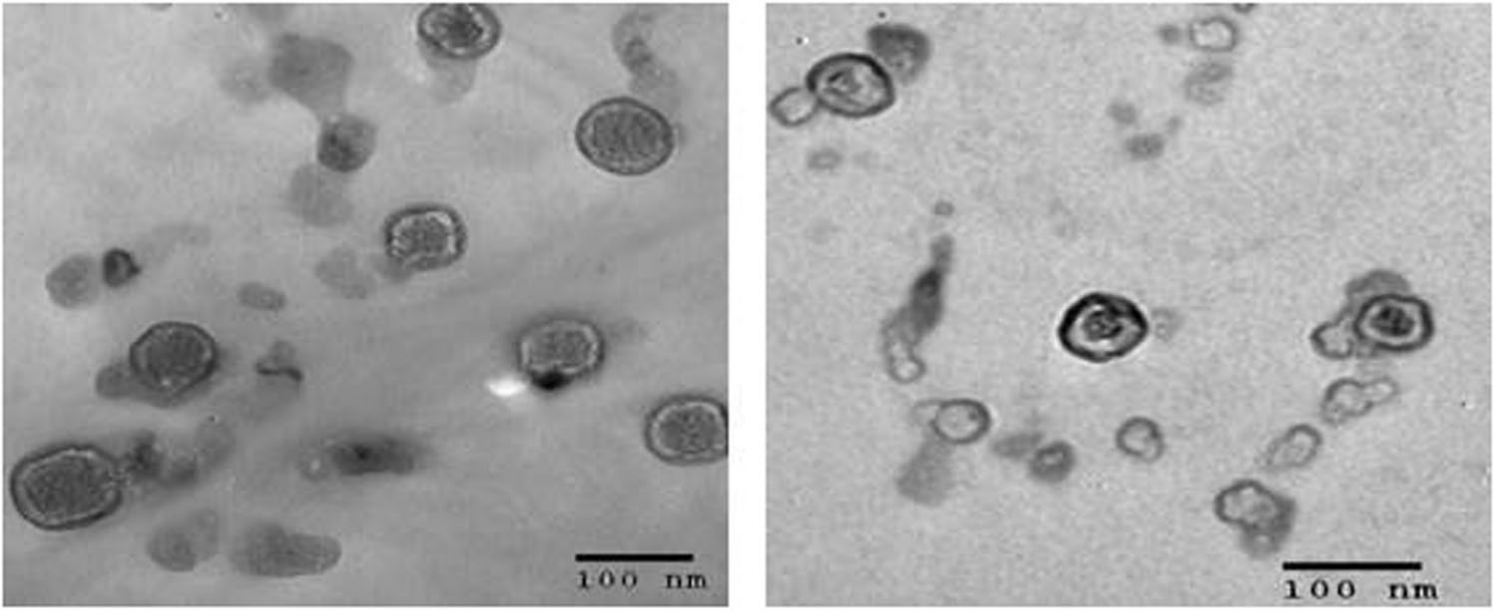
TEM images of acyclovir-loaded niosomes (left) and vancomycin-loaded niosomes (right).

The number-averaged hydrodynamic diameters, polydispersity indices (PDI) and Zeta-potential values for vancomycin- and acyclovir-loaded niosomes are reported in Table 1. The differences in size and size distribution between the two formulations were statistically insignificant, although acyclovir-loaded niosomes had slightly larger diameter (89.7 ± 35 nm *vs*. 53.7 ± 12 nm). Sonication was an important step to reduce the size of the multilamellar vesicles (data not shown). The negative Zeta-potentials measured in all the formulations may be related to the hydroxyl groups in cholesterol, although the acyclovir-loaded niosomes had lower Zeta-potential value. The negative Zeta-potential values observed might provide high physical stability to the dispersions due to electrostatic repulsions between the formed particles. The morphologies observed by TEM for both acyclovir- and vancomycin-loaded niosomes showed dispersed spherical particles with well-defined edges (Fig. 2). Similar to the dynamic light scattering measurements, TEM showed that acyclovir-loaded niosomes had slightly larger diameter than the vancomycin-loaded particles.

### *In vitro* release studies

**Figure 3:**
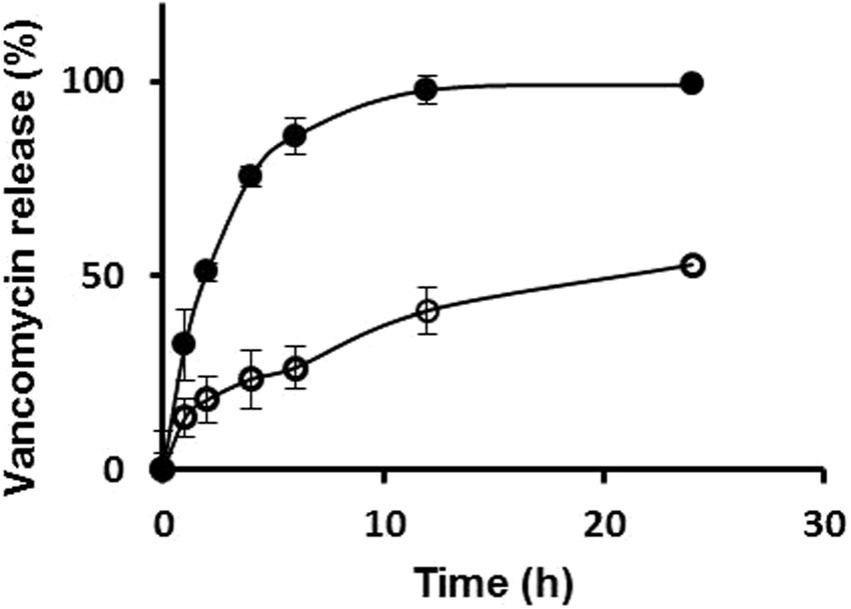
*In* v*itro* release of vancomycin from niosomes (open circles) and vancomycin hydrochloride free drug solution as a control (closed circles) in PBS (pH 7.4). Each value represents the mean ± SD (n = 3).

**Figure 4:**
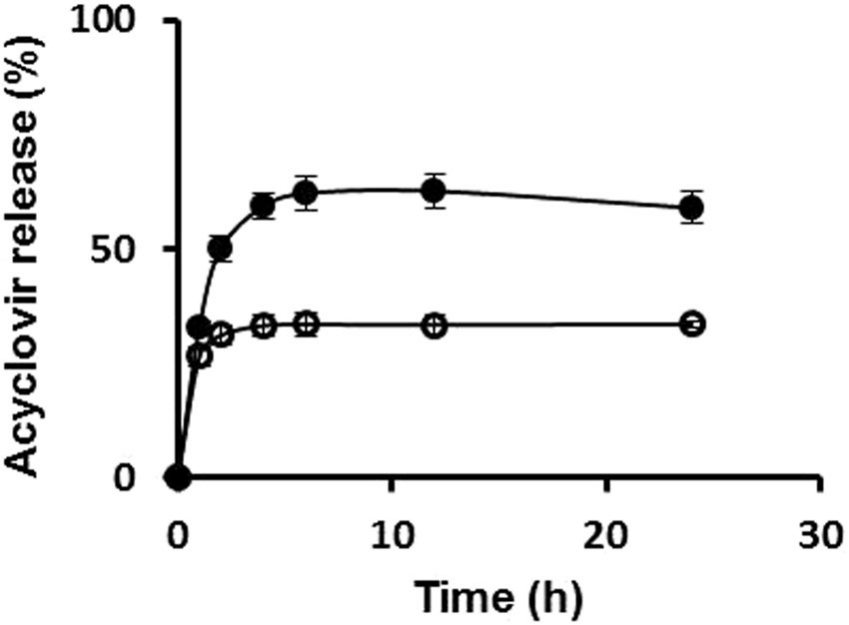
*In vitro* release of acyclovir from niosomes (open circles) and acyclovir sodium free drug solution as a control (closed circles) in PBS (pH 7.4). Each value represents the mean ± SD (n = 3).

The mean cumulative release percentages of each drug that is either free or loaded into niosomes were measured over time (Figs 3 and 4). The percentages of vancomycin release after 2 h were *ca*. 56.2% and 17.2% for vancomycin hydrochloride free drug *versus* the drug incorporated into niosomes, respectively. After 24 h, the percentage of release of vancomycin hydrochloride free drug was *ca*. 91.6% *versus* 43.9% when the drug was loaded into niosomes. In the case of acyclovir sodium, the released percentages of the free drug and drug loaded into niosomes after 2 h were *ca*. 50.1% and 31.1%, respectively. After 6 h, the percentages of release were *ca*. 62.5% and 33.5% for the free drug and the drug loaded into niosomes, respectively. After 24 h, same percentage of release was observed for acyclovir from niosomes and a slight decrease to 58.5% in the case of acyclovir sodium free drug. This decrease is probably due to the partial degradation of the free drug in water after 6 h^9^, which will be confirmed later by thin layer chromatography (TLC) analysis. Overall, the *in vitro* release data demonstrated the rigidity of niosomal membrane and sustained release patterns of drugs loaded into the niosomes. The release profiles of acyclovir sodium and vancomycin hydrochloride from niosomal formulations were studied by fitting the release data to three kinetic models as detailed in the Supplementary Information (Supplementary Information, Table S2). The results indicated that the best kinetic model that fits the release of drugs from niosomes is Higuchi model (the highest regression coefficient (R^2^)). The release rates of vancomycin and acyclovir from niosomes followed a Higuchi, Fickian diffusion^25, 26^.

### Compatibility studies: FT-IR, DSC and percentage of transmittance

**Figure 5:**
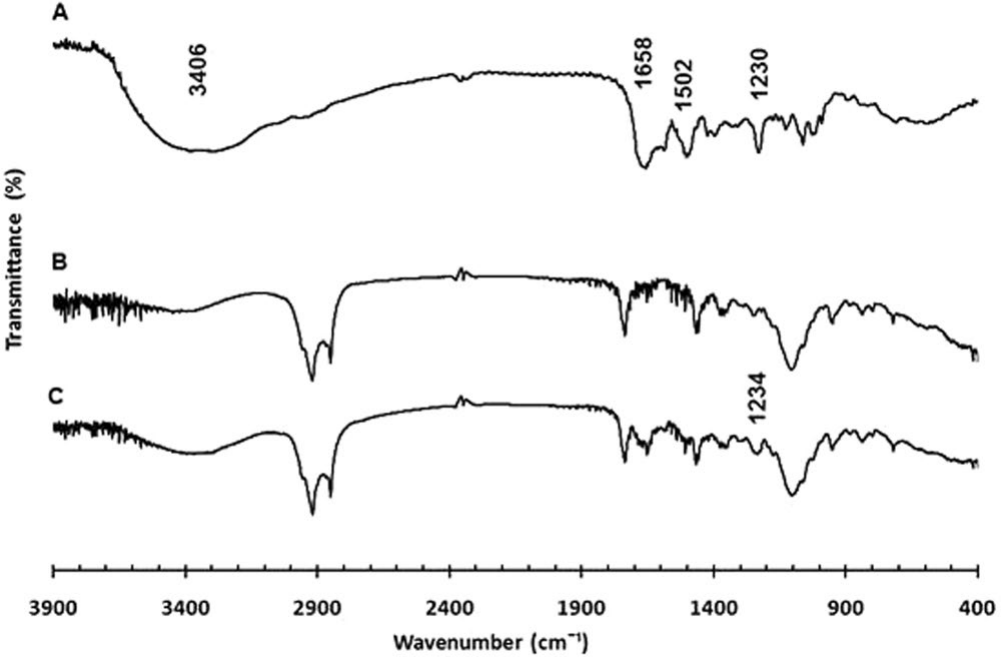
FT-IR spectra of vancomycin hydrochloride free drug (**A**), unloaded niosomes (**B**) and vancomycin-loaded niosomes (**C**).

**Figure 6:**
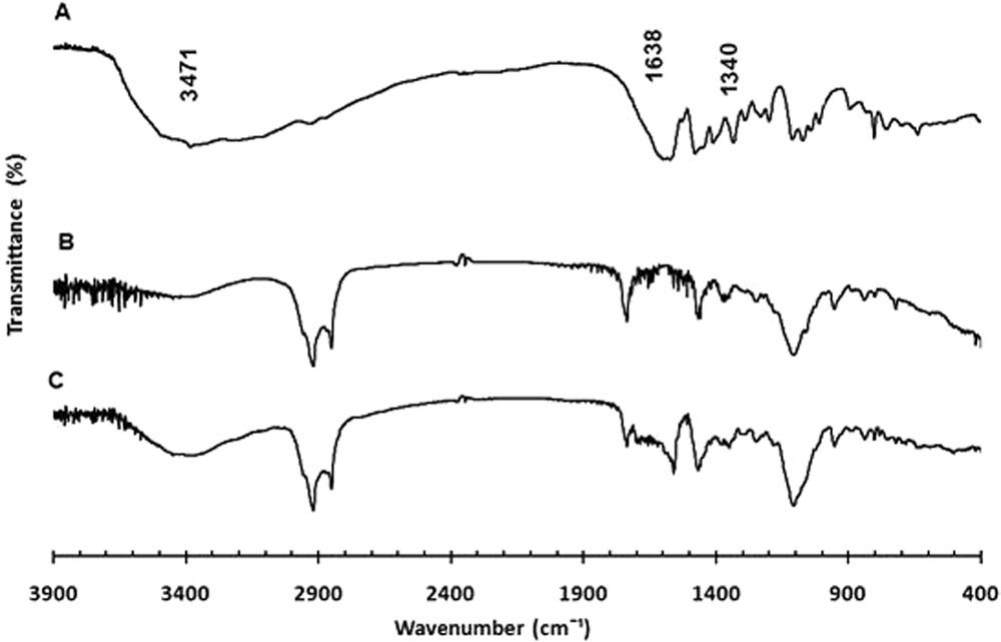
FT-IR spectra of acyclovir sodium free drug (**A**), unloaded niosomes (**B**) and acyclovir-loaded niosomes (**C**).

**Figure 7:**
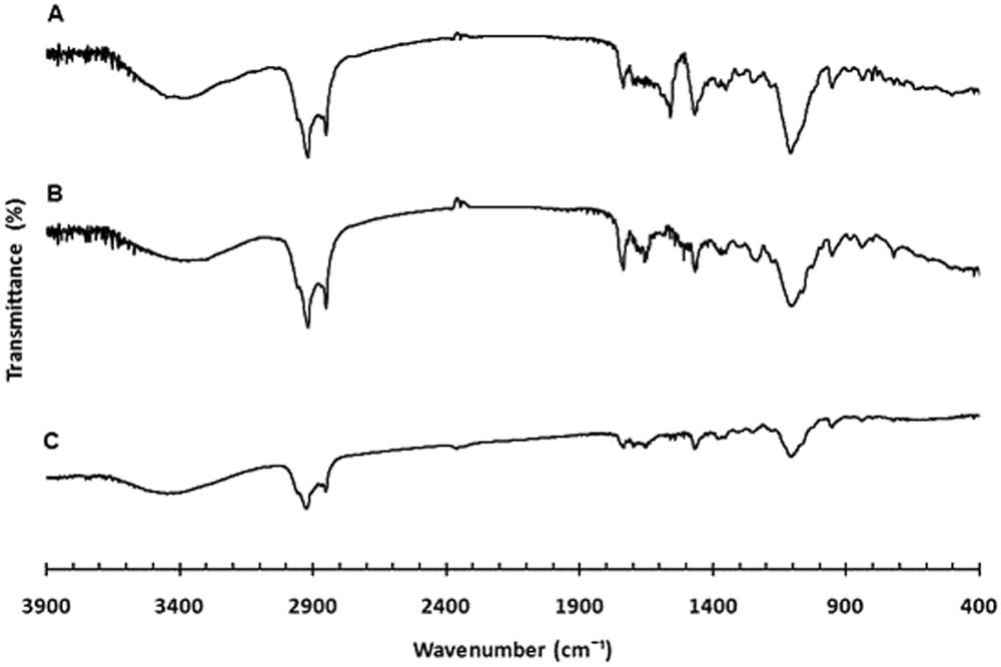
FT-IR spectra of acyclovir-loaded niosomes (**A**), vancomycin-loaded niosomes (**B**), and mixture of acyclovir- and vancomycin-loaded niosomes (**C**).

*Infrared spectroscopy*: The Fourier Transformed-Infrared Spectroscopy (FT-IR) spectra of cholesterol, Span^®^ 60, Tween^®^ 40, vancomycin, acyclovir, as free drugs or loaded separately into niosomes, and spectra of mixed vancomycin- and acyclovir-loaded niosomes have been recoded (Figs 5, 6 and 7 and Supplementary Information, Figures S1–S3). Spectrum of vancomycin hydrochloride showed characteristic peaks at 3406 cm^−1^ of the hydroxyl stretching, 1658 cm^−1^ of the C=O stretching, 1502 cm^−1^ of the C=C, and 1230 cm^−1^ of the phenolic hydroxyl groups (Fig. 5)^27^. FT-IR spectrum of acyclovir showed characteristic bands at 3471 cm^−1^, 1638 cm^−1^ and 1340 cm^−1^, which correspond to N–H stretching, C=O stretching and C–N stretching, respectively (Fig. 6)^28^. Niosomes had similar patterns for both empty and drug-loaded niosomes. Spectra of vancomycin- and acyclovir-loaded niosomes exhibited mainly the peaks of niosomes with few overlapping peaks from vancomycin and acyclovir, respectively, and a shift of the phenolic peak of vancomycin to 1234 cm^−1^ was also observed, which indicate the incorporation of drugs into the niosomes (Figs 5 and 6)^27, 29^. Spectra of mixed niosomal formulations showed also similar pattern to the unloaded niosomes, without the appearance of new peaks in the fingerprint region, which demonstrate the compatibility between the mixed niosomal dispersions (Fig. 7). The possible interactions between the two drugs could not be studied on the IR spectrum of the physical mixture of vancomycin hydrochloride and acyclovir sodium due to peak overlapping in the absorption region of the functional groups (*i*.*e*. OH, NH, CN and C=O) of both drugs (Figure S4).

#### Differential scanning calorimetry (DSC) analyses

**Figure 8:**
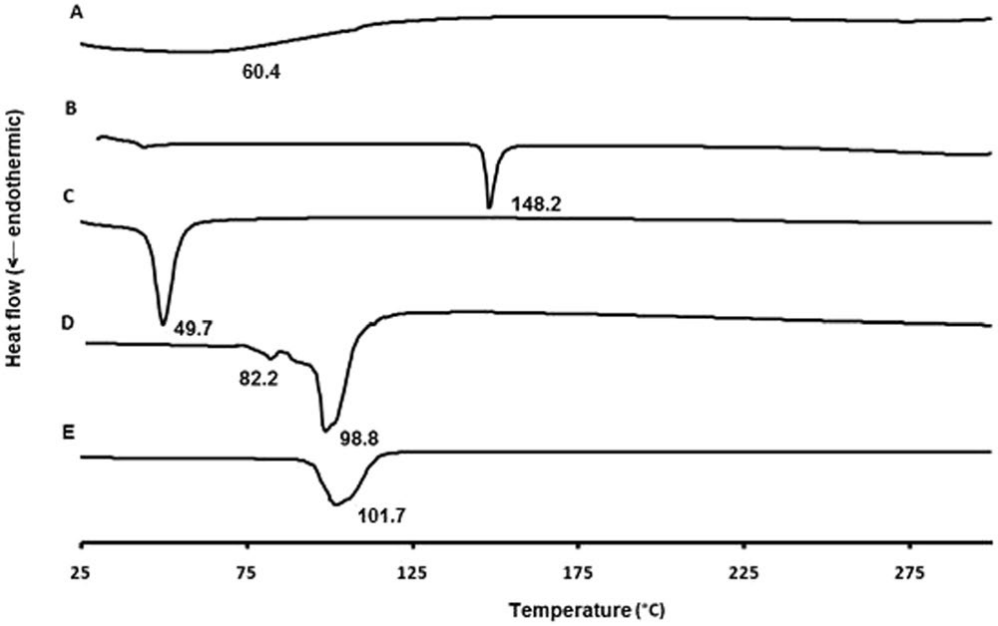
DSC thermograms of vancomycin hydrochloride pure drug powder (**A**), cholesterol (**B**), Span^®^ 60 (**C**), unloaded niosomes (**D**) and vancomycin-loaded niosomes (**E**).

**Figure 9:**
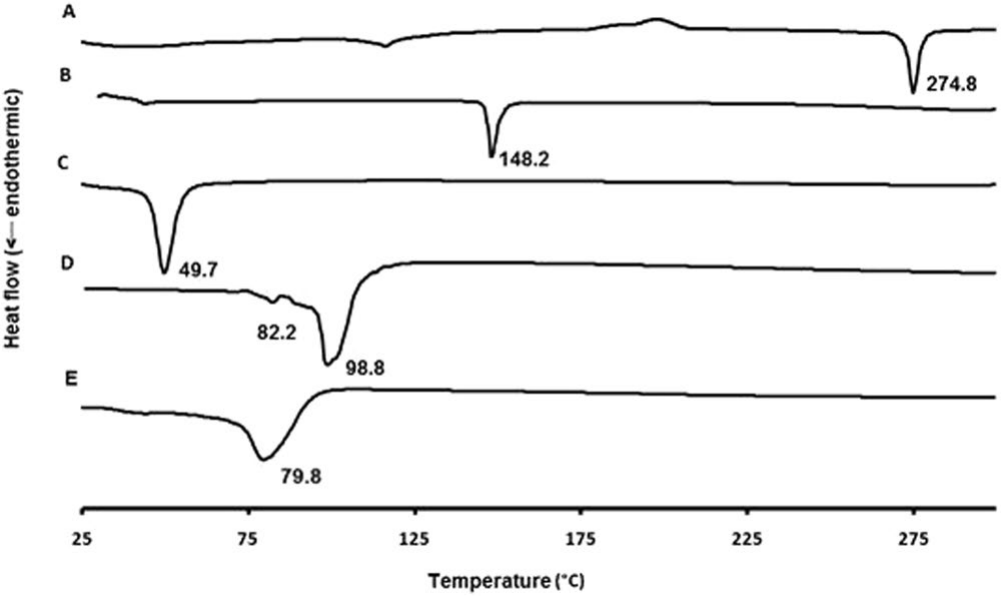
DSC thermograms of acyclovir sodium pure drug powder (**A**), cholesterol (**B**), Span^®^ 60 (**C**), unloaded niosomes (**D**) and acyclovir-loaded niosomes (**E**).

**Figure 10:**
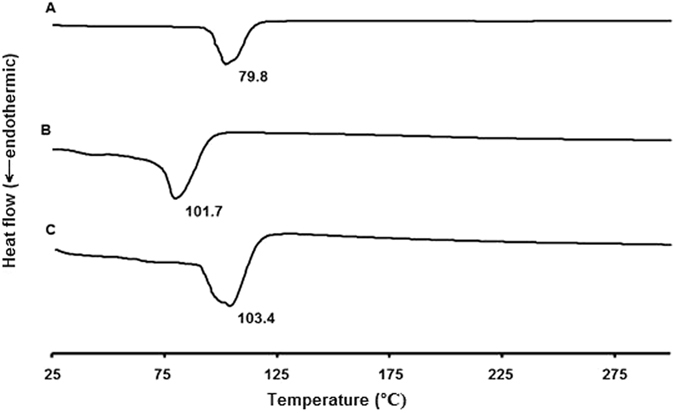
DSC thermograms of acyclovir-loaded niosomes (**A**), vancomycin-loaded niosomes (**B**) and acyclovir- and vancomycin-loaded mixed niosomes (**C**).

DSC thermograms for pure samples of acyclovir sodium, vancomycin hydrochloride, cholesterol, Span^®^ 60, physical mixture of each drug with cholesterol and Span^®^ 60 at a molar ratio of 0.9:2:1 (similar to the ratio in the formulations) were recorded. In addition, thermograms of acyclovir- and vancomycin-loaded niosomes (separate and mixed) were examined (Figs 8, 9 and 10 and Supplementary Information, Figures S5 and S6). Thermogram of vancomycin hydrochloride showed a broad peak at 60 °C (amorphous state)^30^, whereas a narrow sharp endothermic peak at 274.8 °C that corresponds to the melting of acyclovir sodium appeared in the thermogram of acyclovir sodium (Figs 8 and 9)^28, 31^. Physical mixture of acyclovir sodium or vancomycin hydrochloride and different niosomal components showed nearly the same thermal behavior as the individual components (Supplementary Information, Figures S5 and S6). It was observed that when Span^®^ 60 presents in the physical mixture, only a sharp endothermic peak for Span^®^ 60 can be seen at the same position, whereas small peaks were observed for other materials in the mixture^32^. Thermograms of the physical mixtures demonstrate that there were no interactions between the drugs and other materials in the solid state.

In the thermogram of the blank niosomes, cholesterol peak was shifted from 148.2 to 98.8 °C and peak of Span^®^ 60 was also shifted from 49.7 to 82.2 °C, which is due to the interactions between the surfactants and cholesterol to form the niosomes^33^. The disappearance of the characteristic peaks of each drug in the thermograms of the drug-loaded niosomes and appearance of the same pattern of the unloaded niosomes indicate the incorporation of drugs into the niosomes (Fig. 10)^29^. This behavior has been explained previously by the fact that drugs exist in the amorphous phase and homogeneously and molecularly dispersed in the nanoparticle matrix and suggest interactions with the niosomal components^29, 34^. There was also a small shift of cholesterol peak in the drug-loaded niosomes due to the incorporation of the drugs into the niosomes^33^. DSC thermogram of a mixture of both niosomal formulations demonstrated a similar thermal diagram to the unloaded niosomes without appearance of new peaks or peaks of both drugs, and, thus indicating the physicochemical compatibility between the various components. On the contrary, on the DSC thermogram of the physical mixture between the two drugs, the melting peak of acyclovir at 274.8 °C disappeared, and new endothermic peaks could be observed at 55.8 °C, 113.3 °C, and 265.4 °C, which might be due to physicochemical incompatibilities between the two drugs (Supplementary Information, Figures S7 and S8).

#### Percentage of transmittance

Incompatibilities due to the difference in pH occur after mixing two solutions of acyclovir sodium and vancomycin hydrochloride. After mixing both solutions, measured pH of the final solution was 8–10.5, depending on the drugs concentrations (Supplementary Information, Table S1). At this high pH, unionization and precipitation occur for vancomycin. On the contrary, the pH of the mixed niosomal solutions ranged from 7.5–7.9, which is close to the physiological pH and might slightly lower the change in the ionization degrees of the drugs. It is worth mentioning that entrapment and protection of drugs inside the niosomes have the major role in preventing precipitation and degradation of vancomycin and acyclovir because solubilities of both vancomycin and acyclovir decrease at neutral pH. For instance, attempts in literature to prepare vancomycin hydrochloride in buffers of neutral pH (*e*.*g*. phosphate-buffered saline (PBS), pH 7.5) result in drug precipitation at high concentrations^19, 35^. The same trend of low solubility at neutral pH exists for acyclovir^36^. In addition, amphoteric vancomycin may react with acids or bases and thus resulting in various forms of incompatibilities.

**Figure 11:**
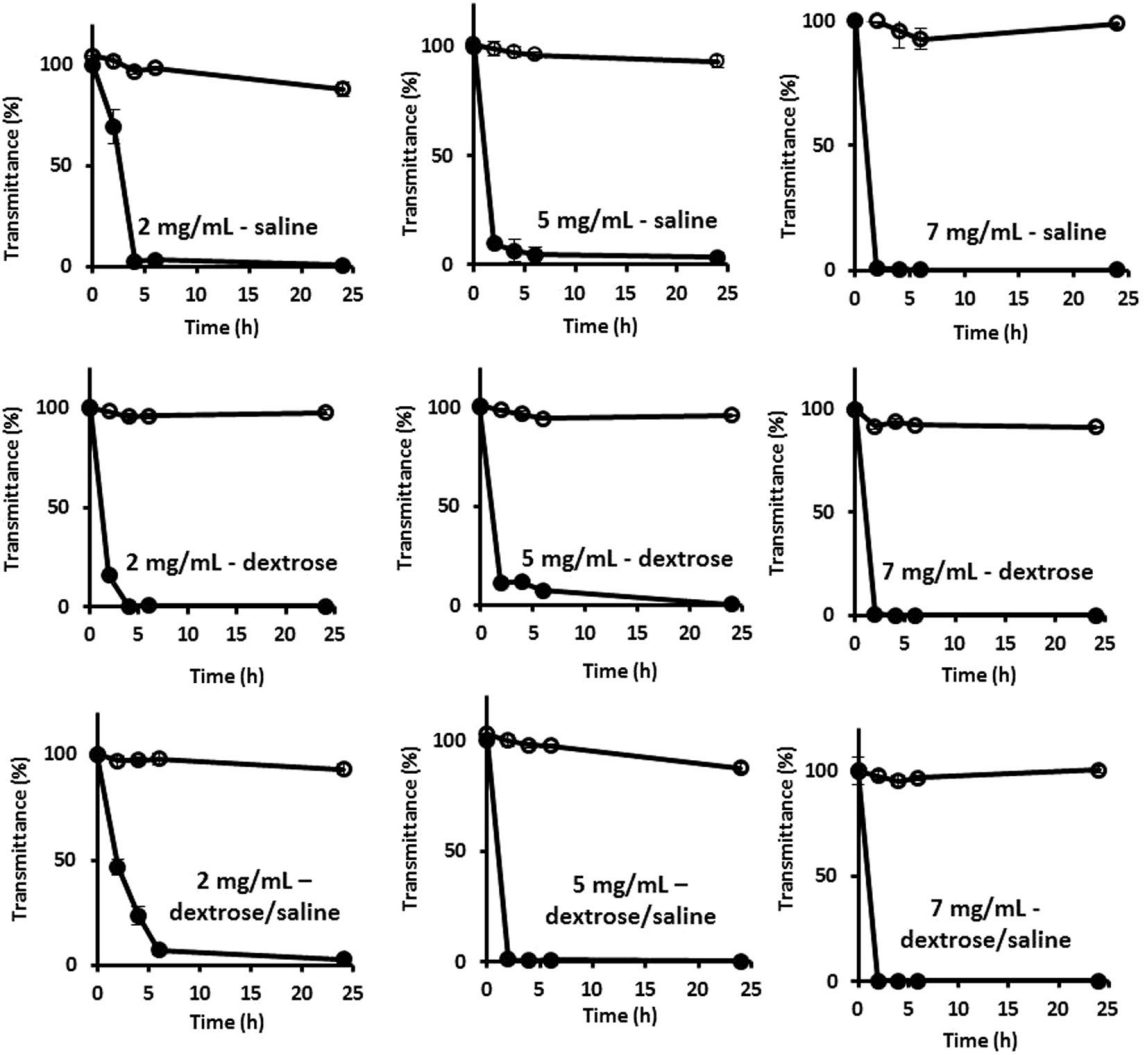
The percentages of transmittance measured by UV–Visible double beam spectrophotometer for mixtures of vancomycin- and acyclovir-loaded niosomes (open circles) and between vancomycin hydrochloride and acyclovir sodium free drug solutions (closed circles) in 0.9% normal saline, 5% dextrose and 5% dextrose/0.9% saline at various concentrations (*i*.*e*. 2, 5 and 7 mg/mL). All samples were exposed to light at room temperature (25 °C). Each value represents the mean ± SD (n = 3).

The percentage of transmittance through dispersions can provide an indication on the turbidity that may result from potential physical interactions in solutions (Fig. 11)^37, 38^. Percentage of transmittance was measured using a double beam spectrophotometer in the visible region, where the amount of absorbed light increases and transmittance decreases as the turbidity increases in the medium in which the light pass through. The percentage of transmittance was measured for all samples prepared from mixed solutions of free powdered drugs and mixed niosomal formulations. In the mixed free drug solutions, steep decline in transmittance was observed over the first few hours. In the case of mixed niosomal samples, the percentage of transmittance remained constant or slightly decreased throughout the whole study. Hence, niosomes appear as promising vehicles to prevent interactions between vancomycin and acyclovir in clinical settings. The type of parenteral solutions (saline *vs*. dextrose *vs*. dextrose in saline) did not influence the rate or degree of interactions between vancomycin and acyclovir, whether they are free in solutions or entrapped into niosomes (Fig. 11). In addition, the effects of temperature (refrigerator *vs*. room temperature) and light *vs*. dark conditions were negligible (data not shown).

A confirmatory experiment was carried out to confirm the validity of using the percentage of transmittance for evaluating physical incompatibilities in mixed solutions of free drugs and drugs loaded into niosomes. In these experiments, percentages of transmittance in solutions of mixed vancomycin- and acyclovir-loaded niosomes were measured before purification (*i*.*e*. drugs exist as free in solution and loaded into niosomes). Percentage of transmittance in the mixed solutions of unpurified vancomycin- and acyclovir-loaded niosomes decreased significantly from 100% immediately after mixing to 79%, 60% and 47% after15 min, 6 h and 24 h of mixing, respectively. On the contrary, percentages of transmittance of the mixed purified niosomal solutions that contain the drug protected into the niosomes remained almost constant for 24 h (Fig. 11). The experiments demonstrate that loading of the drugs into the niosomes is crucial to prevent incompatibilities, and thus physical mixing of drugs and niosomes is not enough to prevent drug precipitation. To further confirm the role of niosomes in preventing incompatibilities between the two drugs, 10% Triton was added to the drug-loaded niosomes and empty niosomes, and solutions were observed visually (Supplementary Information, Figure S9). Clear supernatants were formed upon addition of Triton to vancomycin-loaded niosomes or acyclovir-loaded niosomes and the empty niosomes. In the contrast, turbidity appeared in the supernatant that contains a mixture of vancomycin- and acyclovir-loaded niosomes after addition of Triton due to the rupture of niosomes and release of the two drugs that become free in the solution.

### Quantitative assessment of drug degradation

**Figure 12:**
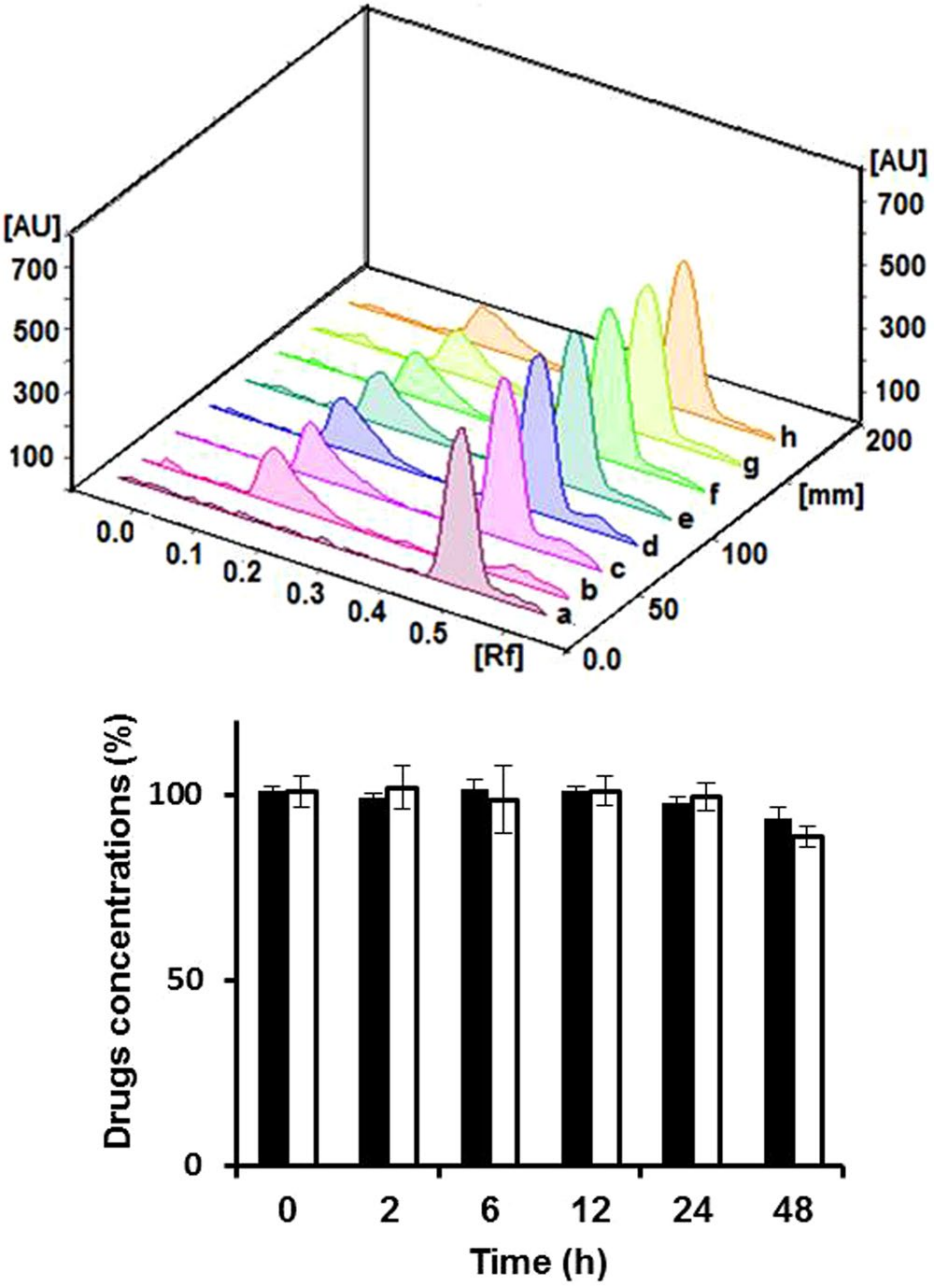
Upper panel is the TLC–scanner 3D diagram of mixtures containing vancomycin- and acyclovir-loaded niosomes (7 mg/mL) from the starting point (**c**) and 2, 6, 12, 24 and 48 h after mixing (**d**–**h**) respectively), in comparison to separate niosomes loaded with acyclovir (**a**) and vancomycin (**b**) at the same concentrations. Lower panel is the concentrations (%) of both drugs in mixtures of vancomycin (white bars)- and acyclovir (black bars)-loaded niosomes from the starting point up to 48 h after mixing as quantified from the areas under the peaks on the TLC diagram in the upper panel.

**Figure 13:**
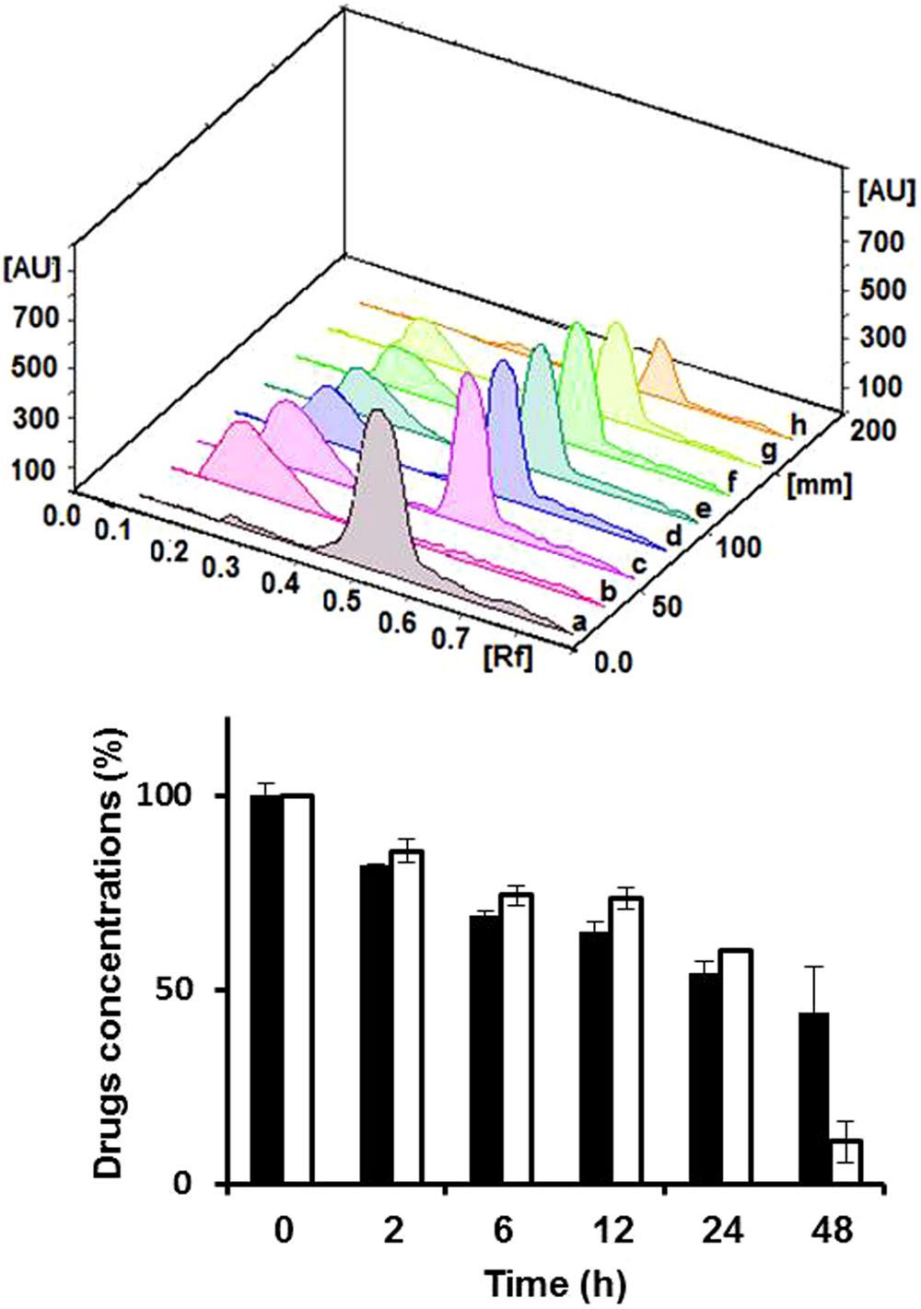
Upper panel is the TLC–scanner 3D diagram of mixtures containing vancomycin hydrochloride and acyclovir sodium free drug solutions (7 mg/mL) from the starting point (**c**) and 2, 6, 12, 24 and 48 h after mixing (**d**–**h**) respectively), in comparison to separate solutions of acyclovir sodium (**a**) and vancomycin hydrochloride (**b**) free drugs at the same concentrations. Lower panel is the concentrations (%) of both drugs in mixtures of solutions of acyclovir sodium (black bars) and vancomycin hydrochloride (white bars) free drugs from the starting point up to 48 h after mixing as quantified from the areas under the peaks on the TLC diagram in the upper panel.

Some incompatibilities result in a physical change (change in color, gas formation or precipitation) that is easily detected. On the contrary, chemical incompatibilities may not be visually detected, where degradation can occur to one or both drugs, and thus, drugs should be quantified *via* using advanced analytical techniques. Thin layer chromatography is a simple and fast chromatographic technique that is used for qualitative and quantitative analysis of organic substances^39^. Separation of mixture of different analytes and studying the progress of specific reaction on the TLC is based on the difference in affinity, and, hence, the adsorption of various analytes onto the stationary phase. Then, the separated spots are detected and quantified *via* measuring the areas under the peaks of the tested analytes. In our study, separate free drug solutions of vancomycin and acyclovir were utilized as controls that have same concentrations of free drugs mixed together or loaded into the niosomes. Mixed drug solutions, free or entrapped into niosomes, were spotted onto the TLC after pre-determined time intervals of incubation and up to 48 h after mixing. In the case of niosomes, Triton was added before spotting the drugs onto the TLC to allow for the rupture of niosomes and release of the entrapped drugs. The chemical interactions between acyclovir sodium and vancomycin hydrochloride free in solutions or loaded into niosomes have been then evaluated (Figs 12 and 13). The 3D chromatograms of mixed niosomal solutions had constant Rf values of 0.47 mm and 0.17 mm for acyclovir sodium and vancomycin hydrochloride, respectively, with nearly the same areas under the peaks, which are related to concentrations of both drugs. Concentrations of both drugs were almost the same over the 48 h and no other spots were detected on the chromatograms (Fig. 12). Although drugs might have been partially released over time from the niosomes, no interactions were observed because precipitation and degradation usually occur at high concentrations. On the contrary, in the case of mixed solutions of free drugs, where the drugs are available at high concentrations in the solutions, the areas under the peaks decreased significantly over time due to degradation of the unprotected free drugs (Fig. 13).

### Preservation of vancomycin antibacterial activity

**Figure 14:**
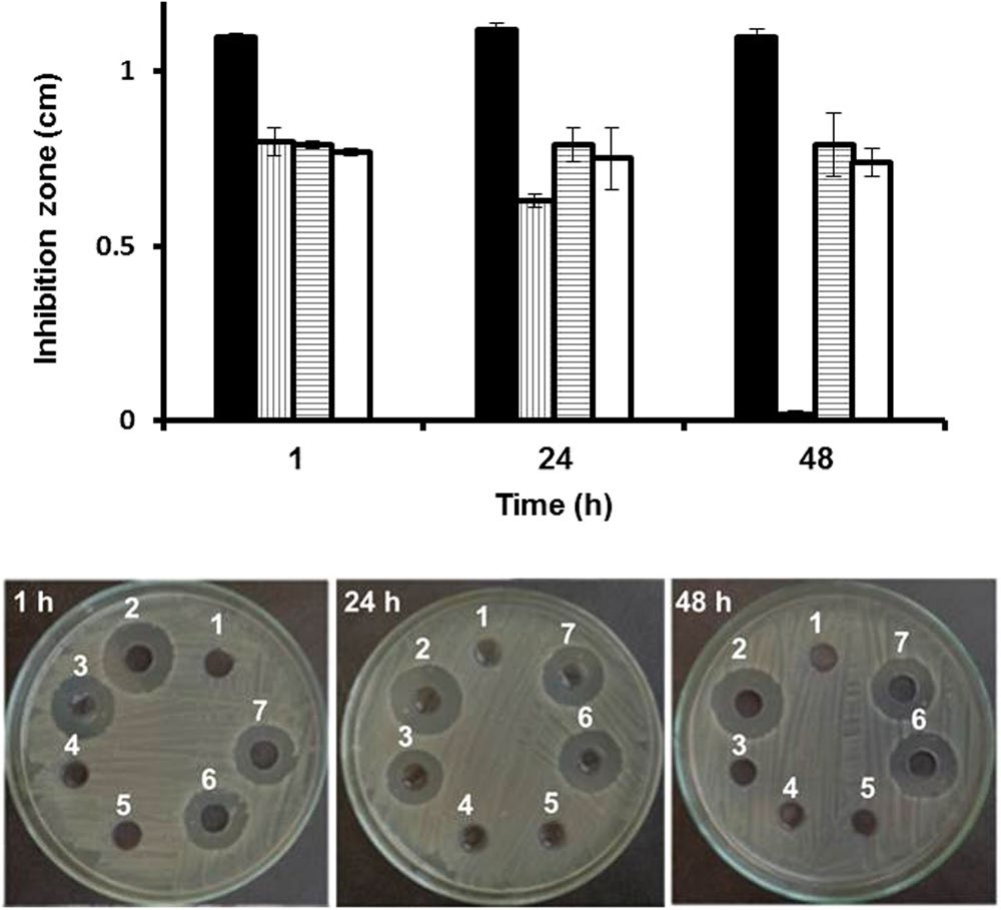
Upper panel indicates the average inhibition zones of methicillin-resistant *Staphylococcus aureus* using agar well diffusion method after addition of vancomycin solution (black bars), vancomycin and acyclovir free drug mixed solutions (vertical stripes), vancomycin-loaded niosomes (horizontal stripes) and vancomycin- and acyclovir-loaded mixed niosomes (white bars). Lower panel demonstrates the micrographs of the inhibition zones of acyclovir sodium solution (**1**), vancomycin hydrochloride solution (**2**), vancomycin hydrochloride and acyclovir sodium mixed solutions (**3**), unloaded niosomes (**4**), acyclovir-loaded niosomes (**5**), vancomycin-loaded niosomes (**6**) and vancomycin- and acyclovir-loaded mixed niosomes (**7**) 1-h, 24-h and 48-h after preparation of solutions or mixing of the tested solutions.

Ability of niosomes to prevent incompatibilities between vancomycin and acyclovir has been confirmed qualitatively (visually and by measuring the percentages of transmittance) and quantitatively (TLC). The purpose of this experiment was to evaluate the ability of niosomes to preserve the antibacterial activity of vancomycin upon mixing with acyclovir (Fig. 14). Various samples were prepared, mixed and incubated for 1, 24 or 48 h before addition to the bacteria, and then antibacterial activities were evaluated using the agar well diffusion method. Methicillin-resistant *Staphylococcus aureus* were incubated with the different formulations for 24 h. As evidenced by the bacterial growth around the formulations, no inhibition zones were observed for the unloaded niosomes, acyclovir free drug solution and acyclovir-loaded niosomes due to the lack of inherent antibacterial activities of these materials. Wide inhibition zones were observed for vancomycin free drug solutions, and slightly decreased by *ca*. 20% in the case of vancomycin-loaded niosomes, which is usually observed for nanoparticles loaded with antibacterial agents due to the sustained release of the encapsulated cargoes over time^27, 31^. The antibacterial activities of vancomycin free drug solution and vancomycin-loaded niosomes were maintained after 48 h of preparation. The antibacterial activity of vancomycin-loaded niosomes did not decrease upon mixing with acyclovir-loaded niosomes, and activity was maintained even after 48 h of mixing, as elucidated by similar inhibition zones throughout the whole study. Acyclovir, when loaded into niosomes could not interact with vancomycin, and thus the antibacterial activity of vancomycin was evident. On the contrary, mixing the free powder solutions of both drugs resulted in *ca*. 27, 43 and 98% decrease of the initial antibacterial activity (*i*.*e*. inhibition zone) of vancomycin, 1, 24 and 48 h after mixing, respectively. The loss of biological activity is due to precipitation and degradation of vancomycin as highlighted earlier in the TLC section and as reported previously in literature^8^. Degradation rate of vancomycin depends on the solution pH, time and temperature, and usually increases in alkaline medium. For instance, at 8 °C in 10 mM ammonium acetate (pH 9), CDP-1 (crystalline degradation product of vancomycin) was formed at a rate of *ca*. 2.7 µg/mL/h^8, 40^. Hence, the reduction in the inhibition zone of free vancomycin/acyclovir mixed solutions is due to the precipitation and degradation of vancomycin. The use of niosomes could preserve the potency and bactericidal activity of vancomycin hydrochloride for 48 h after mixing with the acyclovir-loaded niosomes.

## Discussion

Reconstituted solutions (2–7 mg/mL) of vancomycin and acyclovir have pHs of 5.5–6.5 and 9–11.2, respectively. Mixing both solutions in the same infusion bag results in unionization and precipitation of vancomycin (pH of the mixed solution is 8–10.5), and also degradation of both drugs over time. The higher the concentrations of the free drugs, the higher the differences in pH values of the two solutions, the faster the precipitation occurs after mixing. At concentrations of 2 mg/mL and 5 mg/mL, precipitation occurred after *ca*. 5 h and 1 h, respectively, whereas, immediate precipitation was observed at concentration of 7 mg/mL (Fig. 11).

On the contrary, pH of the mixed niosomal solutions ranged from 7.5–7.9, which is close neutrality due to entrapment of drugs into the cores of niosomes. Niosomes loaded separately with vancomycin and acyclovir could isolate the drugs within the niosomal nanostructures, released the drugs slowly over time and allowed the preparation of solutions of small infusion volumes of appropriate pH, and thus, no precipitation or degradation was observed over 48 h. At a concentration range from 2–7 mg/mL of drugs loaded separately into niosomes, no precipitation was observed over 48 h (qualitatively by measuring the % of transmittance and quantitatively by TLC). Although 7 mg/mL is the highest used therapeutic concentration for both drugs, the maximum concentrations of drugs that could be loaded into niosomes (*i*.*e*. 10 mg/mL) have been also tested and no precipitation was observed over 48 h as evidenced from the constant % of transmittance (data not shown). In addition, the % of transmittance of the mixed drug-loaded niosomal solutions was tested over 72 h and no change in the transmittance was observed (data not shown). Percentage of transmittance in the mixed solutions of unpurified vancomycin- and acyclovir-loaded niosomes decreased significantly over time, as compared to the purified niosomes, which indicate that entrapment of drugs inside the niosomes is essential to circumvent physicochemical incompatibilities between the two drugs.

In addition to preventing drug incompatibilities, loading the drugs separately into the niosomes could maintain the bactericidal activity of vancomycin, as evidenced by growth inhibition of the bacteria (*i*.*e*. methicillin-resistant *Staphylococcus aureus*) incubated with niosomes loaded separately with the two drugs. On the contrary, mixing vancomycin and acyclovir free drug solutions completely abolished the antibacterial activity after 48 h of mixing.

In this study, nanocarriers were utilized, for the first time, to prevent clinically significant drug incompatibilities. The ability of niosomes to prevent incompatibilities between vancomycin and acyclovir has been confirmed qualitatively (visually and by measuring the percentages of transmittance) and quantitatively (TLC). In addition, the use of niosomes could preserve the potency and bactericidal activity of vancomycin hydrochloride for 48 h after mixing with the acyclovir-loaded niosomes. The 48 h duration is much longer than the time needed to keep the two drugs mixed prior to intravenous administration. This study opens a venue for utilizing nanomaterials in preventing drug incompatibilities in clinical settings, aiming at reducing adverse reactions, cost and hospitalization period, and improving patient compliance and therapeutic outcomes. This is expected to expand to a whole new spectrum of applications of nanomaterials in the near future.

## Materials and Methods

### Materials

Vancomycin hydrochloride as lyophilized powder for injection was purchased from Mylan N. V. (Hertfordshire, UK). Acyclovir sodium salt as lyophilized powder for injection was purchased from Wellcome (London, UK). Tween^®^ 40, Span^®^ 60 and cholesterol were purchased from Sigma-Aldrich (St. Louis, MO). Methanol and chloroform were purchased from El-Nasr Pharmaceutical Co. (Cairo, Egypt). Spectro/Por membranes (molecular weight cut-off 12–14 kDa) were purchased from Spectrum Medical Industries, Inc. (Laguna Hills, CA). Parenteral solutions of 0.9% sodium chloride, 5% dextrose and 5% dextrose in 0.9% sodium chloride were obtained from Otsuka Pharmaceutical Co. (Cairo, Egypt). Triton X-100 was purchased from Park Scientific Limited (Northampton, UK). All other used reagents were of analytical grades.

### Methods

#### Preparation of niosomes

Niosomes were prepared by lipid film hydration method. Briefly, cholesterol, Span^®^ 60 and Tween^®^ 40 (2:1:1 molar ratio) were dissolved in 3 mL chloroform. Chloroform was then evaporated under reduced pressure using a rotary evaporator (Buchi 200, BÜCHI Labortechnik AG, Flawil, Switzerland) at 45 °C. The formed lipid film was hydrated with 5 mL of water (45 °C) to form blank niosomes or with 5 mL aqueous solutions of various concentrations of acyclovir sodium and vancomycin hydrochloride to form acyclovir- and vancomycin-loaded niosomes, respectively, after shaking in a water bath for 30 min at 45 °C. Unloaded drugs were separated from the loaded niosomes by centrifugation at 14,000 rpm for 60 min at 4 °C. Niosome pellets were then washed with distilled water followed by centrifugation at 14,000 rpm for 60 min.

#### Dynamic light scattering and Zeta-potential measurements

The mean hydrodynamic diameters and size distributions (PDI) of freshly prepared niosomal dispersions in water (after 100-fold dilution and 2 min of sonication) were measured using a Zetasizer Nano ZS instrument (Malvern Instruments, Worcestershire, UK) equipped with a backscattered light detector operating at 173°. The CONTIN program was used to extract size distributions from the autocorrelation functions. The Zeta-potential values were determined by laser Doppler anemometry using Malvern Zetasizer Nanoseries ZS.

#### Entrapment efficiency

Freshly prepared niosomal dispersions were centrifuged at 14,000 rpm for 1 h at 4 °C using a cooling centrifuge. The formed niosomal pellets were washed and centrifuged, and 10 mL of methanol was added. Both acyclovir sodium and vancomycin hydrochloride were quantified spectrophotometrically at *λ*_max_ of 253 nm and 281 nm, respectively. The concentrations of drugs in the supernatants were also determined. The encapsulation efficiency (%) was calculated according to the following equation:

$${\rm{Encapsulation}}\,{\rm{efficiency}}\,( \% )=\frac{{\rm{amount}}\,{\rm{of}}\,{\rm{drug}}\,{\rm{encapsulated}}\,{\rm{into}}\,\mathrm{niosomes}\,}{{\rm{amount}}\,{\rm{of}}\,{\rm{drug}}\,{\rm{initially}}\,\mathrm{added}\,}\times 100$$

#### Transmission electron microscopy

A drop of niosomal dispersion (equivalent to 1 mg/mL of vancomycin or acyclovir) was placed onto a carbon-coated copper grid for 2 min. Then, excess fluid was removed with a filter paper. A drop of 2% aqueous solution of uranyl acetate was added as a negative staining for niosomes. Samples were visualized and micrographs were taken using a transmission electron microscope (JEOL TEM, Model 100 CX II, Tokyo, Japan) equipped with a digital camera at 80 KV accelerating voltage.

#### *In vitro* drug release

*In vitro* release of acyclovir sodium and vancomycin hydrochloride from niosomes was studied *via* dialysis in a presoaked membrane in PBS at pH 7.4. One mL of niosomal dispersions equivalent to 20 mg of vancomycin hydrochloride or acyclovir sodium were placed in the dialysis bag and dialyzed against 75 mL of PBS (pH 7.4) under shaking at 37 °C. Same conditions were used to study the release of free vancomycin hydrochloride and acyclovir sodium from aqueous solutions (1 mL contains 20 mg vancomycin hydrochloride or acyclovir sodium free powdered drug) as controls. Aliquots of 2 mL were withdrawn at pre-determined time intervals (*i*.*e*. 1, 2, 4, 6, 12 and 24 h), and same volume of fresh buffer was added to replace the withdrawn aliquots and to maintain sink conditions. The collected samples were analyzed at *λ*_max_ of 281 nm and 253 nm for vancomycin hydrochloride and acyclovir sodium, respectively, and PBS was set as the blank.

#### Fourier Transformed-Infrared Spectroscopy (FT-IR)

Infrared spectra of vancomycin hydrochloride, acyclovir sodium, physical mixture of both drugs, cholesterol, Tween^®^ 40, Span^®^ 60, blank niosomes and niosomes loaded with the drugs were recorded using a Nicolet 6700 FT-IR spectrometer (Thermo Fisher Scientific, Waltham, MA). All samples were mixed with potassium bromide (KBr) of spectroscopic grade and compressed into disks using a hydraulic press (15,000 Ib). Niosomal dispersions were tested as thin liquid films on KBr. Samples were scanned from 4000 to 400 cm^−1^.

#### Differential scanning calorimetry (DSC)

DSC analysis using a thermal analyzer (TA-60, Shimadzu, Japan) was performed for samples of vancomycin hydrochloride, acyclovir sodium, cholesterol, Span^®^ 60, Tween^®^ 40, physical mixtures, and for niosomes (empty or loaded with drugs). Samples were examined in a sealed aluminum pans and heated at a constant rate of 10 °C/min up to 300 °C under a nitrogen atmosphere. A similar empty pan was used as a reference. Then, a mixture of vancomycin- and acyclovir-loaded niosomes was examined to detect any changes in the thermal behaviors.

#### UV-Visible double beam spectrophotometer

Formation of precipitate after mixing vancomycin and acyclovir free drug solutions or after mixing niosomes loaded with the drugs could be evaluated by measuring the percentage of transmittance using a double beam spectrophotometer (Shimadzu-50–02, Kyoto, Japan). Mixtures were prepared at various concentrations (2, 5 and 7 mg/mL of each drug) in different types of parenteral solutions (0.9% normal saline, 5% dextrose and 5% dextrose in 0.9% saline), and exposed to normal fluorescent light or kept protected from light. Effect of temperature was also studied by storing the mixtures in a refrigerator or at room temperature. Percentages of transmittance were measured immediately after mixing, and then after 2, 4, 6 and 24 h at a wavelength of 683.2 nm.

#### Thin layer chromatography

Samples were prepared as a binary admixture of acyclovir-loaded niosomes and vancomycin-loaded niosomes at a concentration of 7 mg/mL of each drug, and studied immediately after mixing and after 2, 6, 12, 24 and 48 h. Separate acyclovir- and vancomycin-loaded niosomes were prepared at the same concentrations and studied as controls. Triton (10%) was added to all samples before spotting on the TLC for 1 min to allow for the lysis of niosomes and release of the entrapped drugs to be available for detection. Mixture of free acyclovir sodium and vancomycin hydrochloride solutions at a concentration of 7 mg/mL of each drug was also studied under the same conditions.

TLC analyses were performed on 5 cm × 20 cm aluminum plates coated with 0.2 mm layer of silica gel (60 F_254_). Samples were loaded onto the plate 10 mm from the bottom and 10 mm from the side edges of the plate with a band length of 4 mm. Samples were spotted using linomat V semiautomatic spotting device under continuous drying stream of nitrogen gas. Linear ascending development with 1-butanol: glacial acetic acid: water (20:7.5:12.5 *v*/*v*) as a mobile phase was performed in a glass chamber previously saturated with the mobile phase for 30 min at room temperature (25 ± 1 °C). The optimized development distance and development time were 40 mm and 10 min, respectively. After development, the plates were dried completely and the spots were scanned densitometrically by Camag TLC scanner III (Camag, Muttenz, Switzerland). The TLC scanner was adjusted at a wavelength of 254 nm. All measurements were analyzed by winCATS software. Retention factor (Rf) values, concentrations of the separated compounds and appearance of new spots were determined. All measurements were performed in triplicates.

#### *In vitro* antibacterial activity

Gram positive bacteria, methicillin-resistant *Staphylococcus aureus*, were clinically isolated at the infection control unit (Assiut University Hospital, Egypt). Bacterial suspensions (100 µL) were mixed with Mueller-Hinton agar (20 mL) in sterile Petri dishes (9 cm in diameter) and the agar plates were allowed to solidify. After solidification, wells were made in the agar plates using a pore-maker of size 10 mm and filled with the tested samples. The antibacterial activity of vancomycin-loaded niosomes was measured by determining the zone of inhibition (an indicator for the antibacterial activity) using agar well diffusion method. The effect of mixing both drugs (*i*.*e*. vancomycin and acyclovir) on the antibacterial activity of vancomycin, free or loaded into niosomes, was evaluated. The effect of mixing was evaluated after 1, 24 and 48 h of mixing the various solutions. All samples were prepared at a concentration of 7 mg/mL of each drug, and diluted immediately prior to addition to the agar to a concentration of 50 µg/mL. Plates were incubated at 37 °C for 24 h and the diameters of inhibition zones were measured after subtracting the well diameter from the total inhibition zone diameter using a digital caliber.

## Electronic supplementary material

Supporting Information
