# Supplementary material for: Niosomes: A Strategy toward Prevention of Clinically Significant Drug Incompatibilities
Source: Sci Rep. 2017 Jul 24;7:6340. doi: 10.1038/s41598-017-06955-w (PMC5524940; doi:10.1038/s41598-017-06955-w)
Supplement: Supplementary file 1 — Supporting Information [file 41598_2017_6955_MOESM1_ESM.pdf]

Supplementary Information for:

Niosomes: A Strategy toward Prevention of Clinically Significant Drug Incompatibilities

Hebatallah B. Mohamed[1], Sohair M. El-Shanawany[1], Mostafa A. Hamad[2] and Mahmoud Elsabahy[1, 3-5*]

[1]Department of Pharmaceutics, Faculty of Pharmacy, Assiut University, Assiut 71515, Egypt; [2]Department of Surgery, Faculty of Medicine, Assiut University, Assiut, Egypt; [3]Assiut International Center of Nanomedicine, Al-Rajhy Liver Hospital, Assiut University, Assiut, Egypt; [4]Laboratory for Synthetic-Biologic Interactions, Department of Chemistry, Texas A&M University, College Station, Texas, USA; [5]Misr University for Science and Technology, 6th of October City, Egypt

Correspondence: Mahmoud Elsabahy (mahmoud.elsabahy@chem.tamu.edu)

Tel.: +201000607466

Fax: +20882080711

Kinetic analysis of the *in vitro* release data

The release kinetics of acyclovir sodium and vancomycin hydrochloride from niosomal formulations were studied by fitting the release data to three kinetic models:

Zero-order kinetic: $Q = k_o\ t$

First-order kinetic: $\ln(100\text{-}Q) = \ln_{100}\text{-}k_1t$

Higuchi equation: $Q = k_H\ t_{1/2}$

Where, Q is the amount of drug released at time t, k_o is the zero order release rate constant, K_1 is the first order release rate constant and K_H is the Higuchi release rate constant.

Regression analysis was adopted to compute the constants and squared correlation coefficients (R^2) of the data.

Kinetic analysis of the *in vitro* release of acyclovir sodium and vancomycin hydrochloride from niosomes is demonstrated in **Table S2**. Selection of the kinetic model was dependent on the R^2, where the release kinetics were assigned to the model with the highest value of the calculated correlation coefficient. The release rates of the two drugs from niosomes were dependent on diffusion. Then, the Korsmeyer-Peppas equation was utilized to determine whether the diffusion was Fickian or a non-Fickian, and Fickian diffusion was found for the release of both acyclovir and vancomycin from the niosomes.[1]

Table S1. Measurements of the pH of separate or mixed solutions of vancomycin hydrochloride and acyclovir sodium and separate or mixed solutions of vancomycin- and acyclovir-loaded niosomes. The pH of the blank niosomes is 7.4.

	pH at different concentrations		
	2 mg/mL	5 mg/mL	7 mg/mL
Acyclovir sodium	9	9.5	11.2
Vancomycin hydrochloride	6.5	6.1	5.5
Mixed solutions	8	8.5	10.5
Acyclovir-loaded niosomes	8	8.1	8.1
Vancomycin-loaded niosomes	7.3	6.7	6.6
Mixed niosomes	7.5	7.9	7.9

Table S2. Kinetic analysis of the release data of acyclovir sodium and vancomycin hydrochloride from niosomes.

**Drug-loaded niosomes**	**Zero-order**		**First-order**		**Higuchi-diffusion model**	
	**R^2**	**K_o (mg mL^{-1} h^{-1})**	**R^2**	**K_1 (h^{-1})**	**R^2**	**K_H ($h^{-½}$)**
**Vancomycin**	0.975	1.675	0.988	0.026	0.996	10.312
**Acyclovir**	0.544	0.175	0.547	0.003	0.673	1.308

R^2, correlation coefficient; k_o, zero order release rate constant; K_1, first order release rate constant; K_H, Higuchi release rate constant.

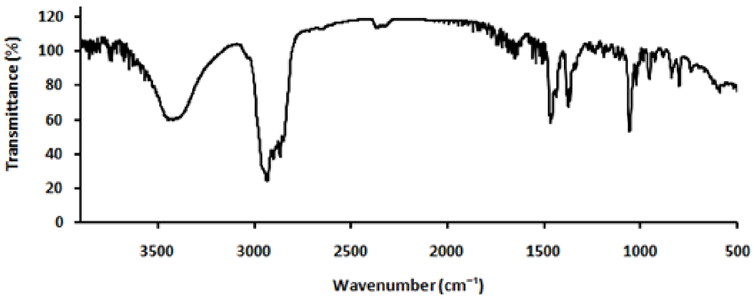

Figure S1. FT-IR spectrum of cholesterol.

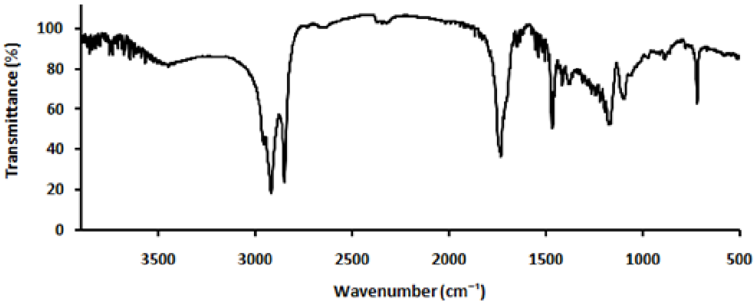

Figure S2. FT-IR spectrum of Span® 60**.**

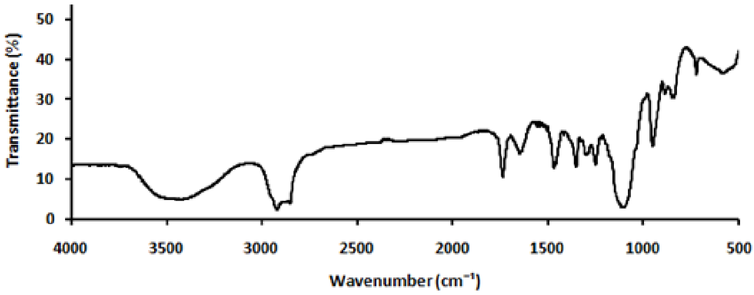

Figure S3. FT-IR spectrum of Tween® 40**.**

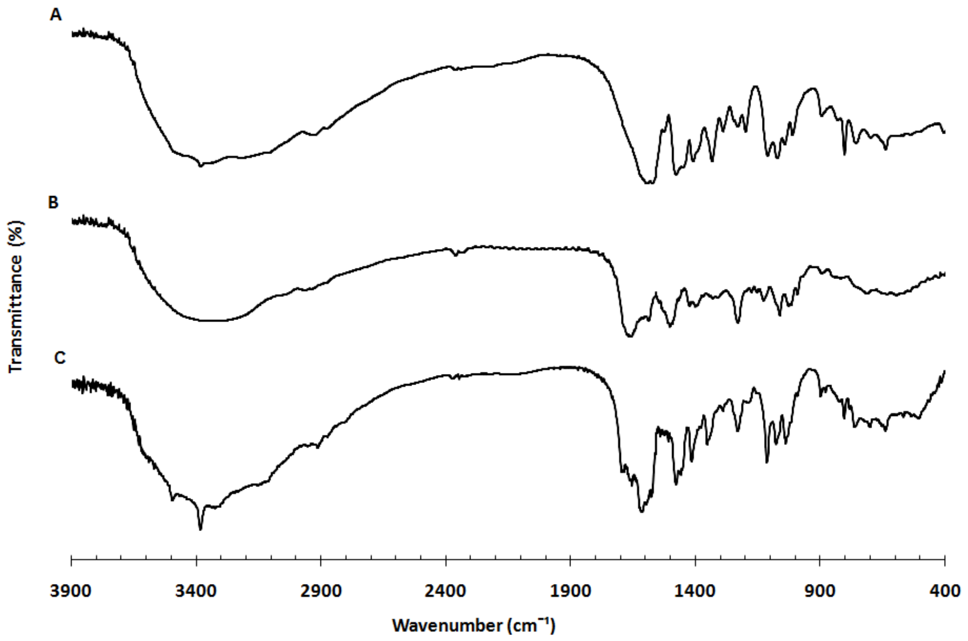

Figure S4. FT-IR spectra of acyclovir sodium free powder (**A**), vancomycin hydrochloride free powder (**B**) and mixed acyclovir sodium and vancomycin hydrochloride powders (**C**).

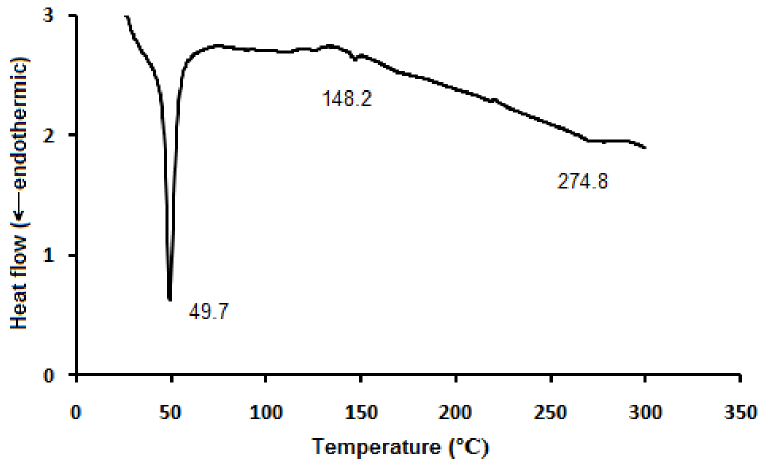

Figure S5. DSC thermogram of a physical mixture of acyclovir sodium, cholesterol and Span® 60.

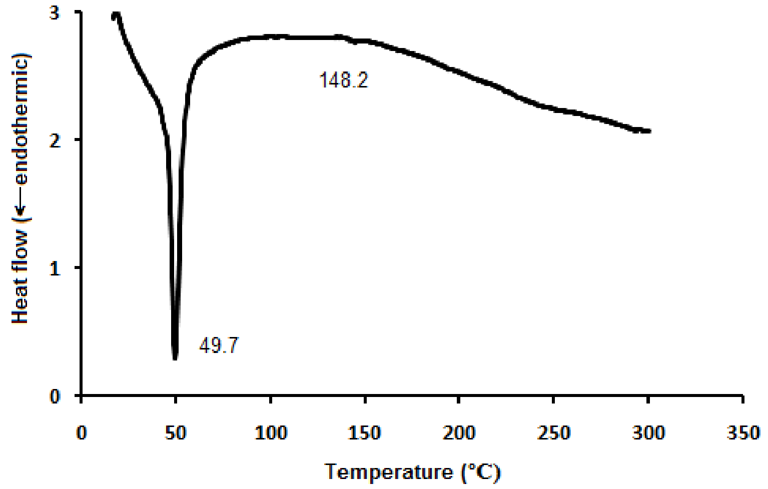

Figure S6. DSC thermogram of a physical mixture of vancomycin hydrochloride, cholesterol and Span® 60.

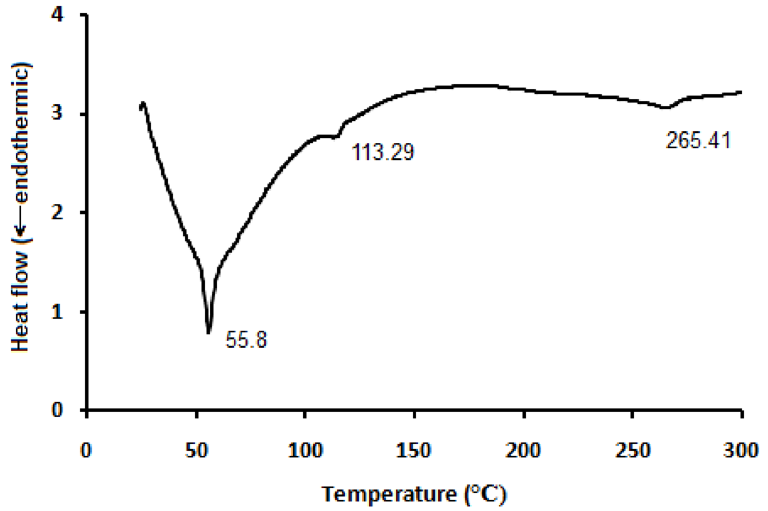

Figure S7. DSC thermogram of a mixed powder of acyclovir sodium and vancomycin hydrochloride free drugs.

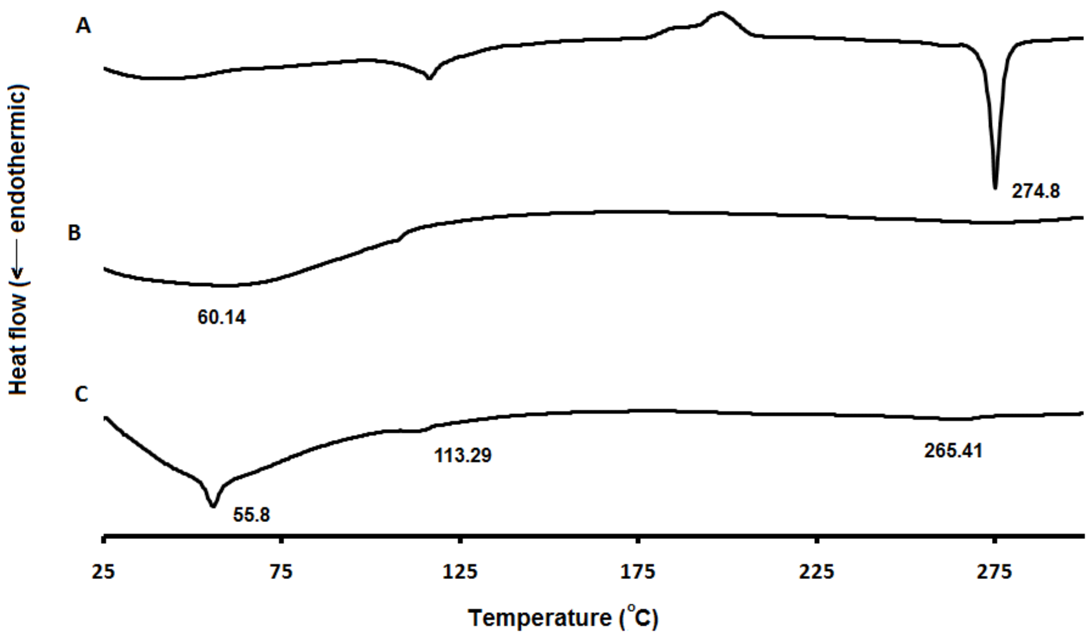

Figure S8. DSC thermograms of acyclovir sodium (**A**), vancomycin hydrochloride (**B**) and a mixed powder of both free drugs (**C**).

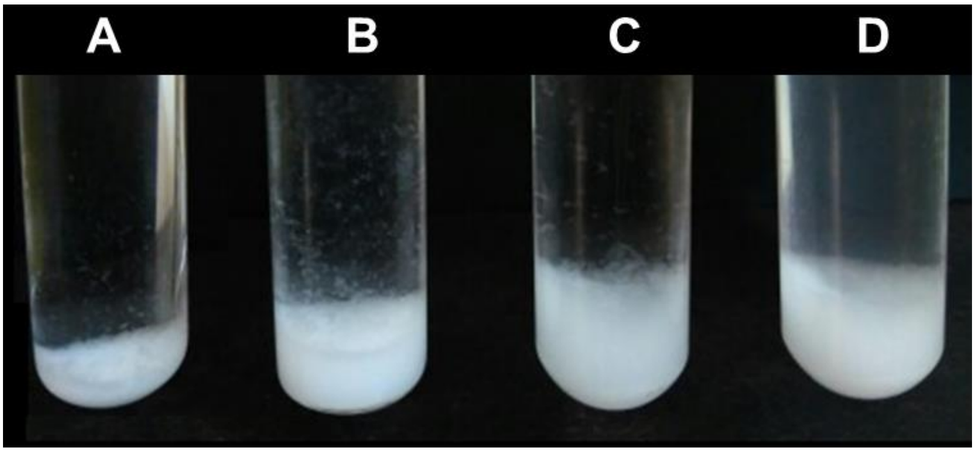

Figure S9. Clear solutions (*i.e.* supernatants) were formed upon addition of 10% Triton to acyclovir-loaded niosomes (**A**), vancomycin-loaded niosomes (**B**) and unloaded niosomes (**C**). On the contrary, turbidity appeared in a mixture of vancomycin- and acyclovir-loaded niosomes (**D**) after addition of 10% Triton due to the rupture of niosomes and release of the two drugs that become free in the solution.

References

1 El-Badry, M. & Fetih, G. Preparation, charactarization and anti-inflammatory activity of celecoxib chitosan gel formulations. *J DRUG DEL SCI TECH* **21**, 201-206 (2011).
